# Evolutionary Story of the Low/Medium-Affinity IgG Fc Receptor Gene Cluster

**DOI:** 10.3389/fimmu.2019.01297

**Published:** 2019-06-06

**Authors:** Julien Lejeune, Guillaume Brachet, Hervé Watier

**Affiliations:** ^1^EA 7501 GICC Université de Tours, Tours, France; ^2^CHRU de Tours, Tours, France

**Keywords:** FcgR, genetics, mammals, evolution, recombination, evolution, retroelement

## Abstract

Low/intermediate affinity Fc-gamma receptors (FcγR) are crucial for the recognition of immune complexes and IgG-sensitized microorganisms by phagocytic and cytotoxic effector cells. In all mammalian species studied so far, their genes are clustered in a single locus. However, this locus differs between humans and mice, both in the number of genes and the structure/function of the encoded receptors. We show that murine fcgr3 evolved through several steps into FCGR2A, its ortholog, which is specific to primates. One of these steps was the insertion of a retroviral element bringing a new intracellular exon comprising a non-canonical ITAM motif. We also show that the fcgr3-hspa6-fcgr4-fcgr2b module in mammals that has evolved in a FCGR2A-HSPA6-FCGR4-FCGR2B module in primates, was subsequently duplicated in apes through a Non-Allelic Homologous Recombination (NAHR), giving birth to FCGR2C, a hybrid gene between FCGR2B and FCGR2A. The FCGR4 duplication, which occurred simultaneously, eventually resulted in the emergence of FCGR3B, while FCGR3A remained the true FCGR4 ortholog. FCGR2C and FCGR3B, markers of this NAHR, are present in gorillas and chimpanzees, whereas they are absent in orangutans and more distant primates, such as gibbons and macaques. These data need to be taken into account when testing IgG-based therapies in animal species.

## Introduction

Receptors for the Fc portion of IgG (FcγR) play an important role by connecting innate and adaptive immunity. This is particularly true for receptors of low/intermediate affinity which are specialized in the recognition of immune complexes and IgG-sensitized microorganisms or target cells by phagocytic and cytotoxic effector cells. In all mammalian species studied so far, all the genes encoding these low/intermediate affinity FcγRs are clustered in a single locus. This clustering probably allows gene plasticity and rapid evolution to maintain adequate binding of the different FcγR to their different ligands (IgG subclasses), which are themselves in continuous evolution, and to maintain the immune functions conferred to the effector cells.

In mice, there are three different genes, gathered on distal chromosome 1: *Fcgr2* and *Fcgr3*, coding for FcγRII and FcγRIII, respectively, and the more recently identified *Fcgr4* coding for FcγRIV (1–3). FcγRIII and FcγRIV, as well as the high affinity FcγRI (gene located on chromosome 3), are associated with the FcR-γ chain, which carries an Immunoreceptor Tyrosine-based Activation Motif (ITAM) required for cell activation (2, 3). FcR-γ KO mice do not express these activating receptors and are deficient in antibody-mediated responses such as antibody-dependent cell-mediated cytotoxicity (ADCC) and antibody-dependent cell phagocytosis (4, 5). By contrast, FcγRII includes an Immunoreceptor Tyrosine-based Inhibitory Motif (ITIM) in its intracellular portion and can inhibit activating receptors when both types of receptors are coengaged (6). Accordingly, mice lacking *Fcgr2* show augmented humoral responses and increased responses to therapeutic antibodies (4, 5).

In humans, there are five genes encoding low/intermediate affinity IgG Fc receptors, clustered on chromosome 1q21-q23: *FCGR2A, FCGR2B, FCGR2C, FCGR3A*, and *FCGR3B*. The situation therefore appears much more complex than in mice, with an increased number of genes, and also by the fact at least three of them (*FCGR2A, FCGR2C*, and *FCGR3B*) are coding for FcγRs displaying properties not existing in mice. *FCGR2A* encodes the activating receptor FcγRIIA, which is described as an equivalent to murine FcγRIII (7), although it does not associate with FcR-γ. Indeed, FcγRIIA possesses its own activation motif in its intracellular (IC) domain, a non-classical ITAM characterized by the presence of 12 residues between the two YXXL boxes, instead of 7 in classical ITAMs. This non-classical ITAM has been associated with differences in intracellular signaling pathways and functional activities (8). *FCGR2C* is a chimeric gene that is the result of an unequal crossing-over between *FCGR2A* and *FCGR2B*, having extracellular domains similar to FcγRIIB and an intracellular ITAM-like motif identical to that of FcγRIIA (9). *FCGR3B* codes for FcγRIIIB, an atypical receptor attached to the cell membrane by a glycosyl-phosphatidylinositol (GPI) anchor and having no intrinsic triggering capability even though it could contribute to cell activation by associating with other FcγRs (10). *FCGR2B* encodes the only ITIM-containing inhibitory receptor, in both human and mice, and is therefore likely the ortholog of murine *Fcgr2*. FcγRIIIA is the only human low/intermediate affinity FcγRs requiring FcR-γ as transduction subunit, like murine FcγRIII and FcγRIV, but sequence analyses suggest that *FCGR3A* could be the human ortholog of murine *Fcgr4* (2).

Little is known about the evolution of the FCGR2/4 gene cluster and the mechanisms which led to the acquisition of FCGR2C and FCGR3B in human. Moreover, despite the functional consequences of the ITAM-like motif, no hypothesis has been proposed to explain its presence in FcγRIIA and FcγRIIC so far.

Amongst non-human primates, FcγRIIA has been identified in macaque, baboon and mangabey (11). Rhesus, cynomolgus and pigtail macaques are also known to express FcγRIIA and IIB (12). As for other mammals, FcγRII is known to be expressed cows, and FcγRIII by cows and pigs, without any further detail as to which isoform (A or B) these receptors correspond to (13).

In order to gain insight into the evolutional history of the FCGR2/4 cluster, we studied the genes coding for low-affinity FcγRs in several mammalian species, including several primates. Nucleotide alignments were performed on the genomes to find low/medium affinity FCGR orthologs, which were amplified using long PCR and further characterized through sequencing and alignment with orthologs. A sequence suggesting a genetic recombination event was found and compared with known viral retroelements. We found that the cluster is composed of 5 genes in humans, chimpanzees and gorillas but by only three in macaques, gibbons, orangutans and other mammals. We also confirm that macaques are characterized by a unique FcγRIIA receptor with an ITAM-like motif, and extend this feature to other primates (14).

## Materials and Methods

### DNA

Genomic DNA from chimpanzee (ECACC No. 89072704), gorilla (ECACC No. 89072703), orangutan (ECACC No. 89072705), gibbon (ECACC No. 86102901) and cynomologous monkey (ECACC No. 90071809) cell lines was purchased from European Collection of Cell Cultures. DNA was extracted from buccal samples of six Gorillas and 8 Orangutans, using Isohelix DNA isolation kit. One additional gorilla DNA was obtained by blood extraction using QIAGEN DNA extraction kit (Qiagen, ref. 51104).

### Identification of the Medium/Low Affinity Loci in Other Mammals

The mammalian genomes were systematically screened for Fcgr2/3 orthologs using BLAST for nucleotidic alignment, with default parameters (15). The contiguous sequences, the versions used and the updated versions where appropriate, are described in (Supplementary Table 1).

### Amplification and Sequence Analysis of *FCGR2A*/*FCGR2B*/*FCGR2C* Genes

Long-PCR was performed with Elongase Amplification System (Invitrogen, ref. 10480010) according to manufacturer procedure, with 1.5 mM of MgCl_2_. Primers were designed on highly conserved coding sequences observed between primates in order to amplify genes of interest. Special care was taken in order to design primers with high gene specificity and with sequence compatibility including mammals other than primates when possible. For *FCGR2A* amplification, an 8 kb fragment was obtained using a 2aMfe2-S forward primer located in exon 2 (5′GCTTCTGCAGACAGTCAAACTG3′) and a 2aMfe7-AS reverse primer located in exon 7 (5′TCGGGCAGCCTTCACAGGATC3′). For *FCGR2B* amplification a 7.5 kb fragment was obtained using a 2bMfHse2-S forward primer located in exon 2 (5′CTCCTGTTGCTGGGACACCTG3′) and a 2bMfHse8-AS reverse primer located in exon 8 (5′TGAATAGGTGATTGTGTTCTCAGC3′). For FCGR2C, which is a chimeric gene between *FCGR2A* and *FCGR2B*, a 7.2 kb fragment was obtained using 2bMfHse2-S forward primer and 2aMfe7-AS reverse primer. For gibbon, due to divergence, 2aGibbon-S primer (5′CTTCTGCAGACAGTCAAGCTG3′) was used instead of 2aMfe2-S. For all species, 100 ng of genomic DNA sample were amplified in 20 μL final volume on a Bio-Rad MJ Mini Thermal Cycler with an initial denaturation at 94°C for 5 min, followed by 35 cycles of: 94°C for 30 s, 63.1°C for 30 s, 68°C for 10 min. Amplification was verified by loading PCR products on a 0.8% agarose gel, which was revealed using ethidium bromide. All the PCR products where secondly sequenced using the primers described in Supplementary Table 2.

### Amplification and Sequence Analysis of *FCGR3A* and *FCGR3B* Genes

PCR was performed with Taq Polymerase (Eurobio, ref. GAETAQ004D) following the manufacturer's instructions. Primers were designed as described above. We referred to a genomic alignment to ensure that primers would amplify the intended fragments in all considered species. For co-amplification of *FCGR3A* and *FCGR3B*, a 176 bp product was obtained with a 3Mfe5-S forward primer (5′TTTGGCAGTGTCATCCATCTC3′) and a 3Mfe5(2)-AS reverse primer (5′ATTTGTCTTGAGGGTCCTTGCTCC3′), both located in exon 5. PCR was conducted as described above, except that initial denaturation was followed by 35 cycles of: 94°C for 30 s, 55.7°C for 30 s, 68°C for 10 min. Amplification was verified by loading PCR products on an 8% acrylamide gel, which was revealed using ethidium bromide. All the PCR products where secondly sequenced.

### Bioinformatic Analysis

Sequences were collected in genomic and cDNA databases by similarity searching using the BLAST tools (15), all available on the NCBI website (http://www.ncbi.nlm.nih.gov/). Sequences for endogenous retrovirus were extracted from REPBASE using CENSOR tool (16) available on GIRI web site (http://www.girinst.org/) and from RepeatMasker (http://www.repeatmasker.org/). Multiple alignments for the genomic analysis were done using CLUSTALW (17) followed by visual inspection and manual editing when necessary. The Neighbor-Joining trees were obtained using Kalign algorithm (18).

## Results

### Genesis of *FCGR2A*, a New Activating Receptor Gene in Primates

**Figure 1:**
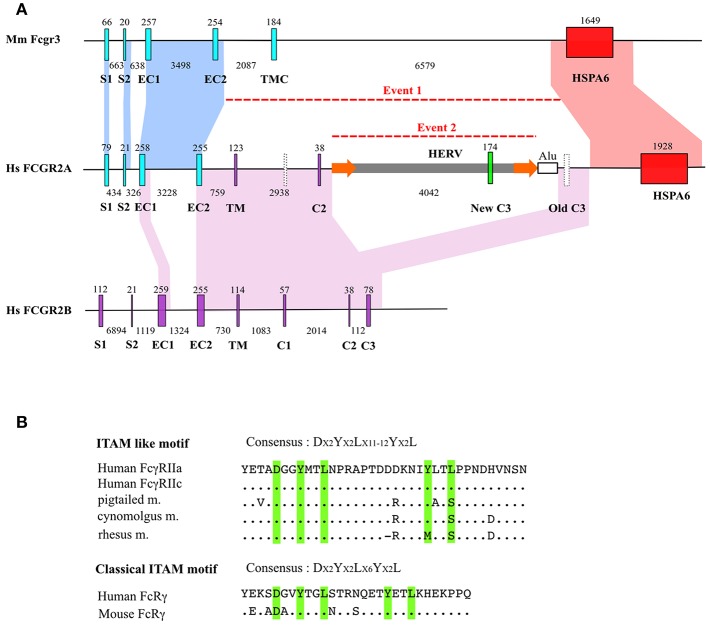
*FCGR2A* genesis. **(A)** Intron/exon organization of mouse *Fcgr3* and human *FCGR2B* and *FCGR2A* genes. Homologous regions (defined by default BLAST E-value) were indicated by areas of identical colors. Major insertion events are indicated by dotted lines. Exons are abbreviated as follows: signal peptide 1 (S1) and 2 (S2), extracellular 1 (EC1) and 2 (EC2), transmembrane (TM) and intracytoplasmic 1 (C1), 2 (C2), and 3 (C3) domains. HSPA6 stands for Heat Shock Protein Family A (Hsp70) Member 6. HERV stands for Human Endogeneous RetroVirus. Exon and intron size are indicated above and below each gene. For clarity purposes, there is no correspondence between nucleotidic size of the represented genetic elements in kb and scale in the figure. Empty vertical bars represent non-coding exons. Flanking LTRs around HERV are shown as orange arrows. **(B)** Comparison of *FCGR2A* ITAM-like motif coding by retroelement with canonical ITAM motif. Dots indicate identity and dashes indicate gaps.

Analysis of human and mouse *FCGR* sequences was performed and confirmed orthology between human *FCGR3A* and mouse *Fcgr4* and between human *FCGR2B* and mouse *Fcgr2b*. Alignment of mouse *Fcgr3-HspA6* segment with human *FCGR2A-HSPA6* showed extensive homology, notably in S1 and S2 exons, from the EC1 to the EC2 exons, including the third intron, and the whole *HSPA6* gene, including upstream and downstream regions (Figure 1A).

These observations confirm an orthologous relationship between *Fcgr3* and *FCGR2A*.

Interestingly, although mouse *Fcgr3* and *Fcgr2b* show no homology except between their EC1 and EC2 exons (data not shown), human *FCGR2A* aligns with *FCGR2B* not only at the level of EC1 and EC2 exons, but also on a large stretch extending for the 5′ part of EC2 to the 3′ part of IC3 of *FCGR2B*. This alignment is interrupted by a ~5 kb segment characterized by long terminal repeats (LTR) suggesting a retroviral origin (Figure 1A). Annotation from Repeatmasker showed that these LTR present high homology with LTR from MER57F, a class I human endogenous retrovirus. No homology with the classical retrovirus genes *gag, pol* and *env* was found in the sequence. Analysis of the internal part of the sequence revealed a high homology with MER34B, a non-autonomous retroelement (Supplementary Table 3). The presence of this element was tested among other mammals by LTR signature to evaluate the age of this *Fcgr3* insertion. Traces of this retroelement were found in *Otolemur garnetii* (Lemuriformes), *Callithrix jacchus* (Callitrichidae), and in *Macaca mulatta* (Cercopithecidae) which revealed that its acquisition is probably specific to primates. A new *FCGR2A* IC3 exon appeared as being encoded by the endogenous retrovirus segment homologous to MER34B. The non-canonical ITAM motif contained in FcγRIIA and FcγRIIC, unique to primates and encoded by the new IC3 exon, possesses a DX_2_YX_2_LX_**12**_YX_2_L sequence in human and chimpanzee FcγRIIA and FcγRIIC, replaced by a DX_2_YX_2_LX_**11**_YX_2_L sequence in cynomolgus monkey (Figure 1B). A nucleotide sequence potentially encoding DX_2_YX_2_LX_**11**_YX_2_L in another composite endogenous retrovirus is located in chromosome 11p14.3, between the *NELL1* and *TMEM16E* genes. This sequence is not associated to any known transcript. The vestigial IC3 exon downstream the endogenous retrovirus, which was homologous to *FCGR2B* IC3, has never been found in *FCGR2A* (or *FCGR2C*) mRNA, even in the EST database.

### Genomic Organization of *FCGR2/3* Cluster in Mammals

#### Segmental Duplication of Cluster *FCGR2/3* in Primates

**Figure 2:**
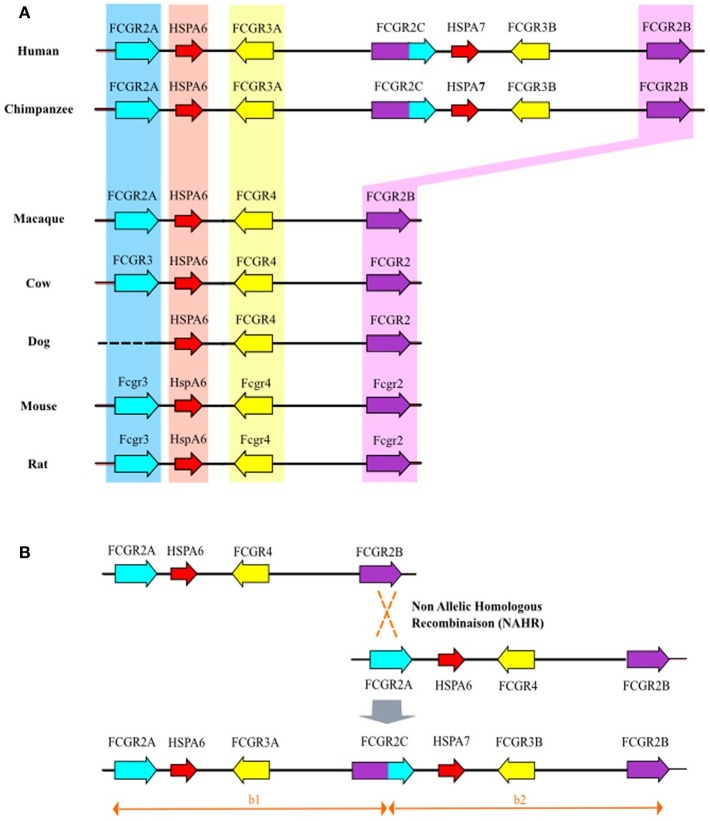
**(A)** Genomic organization of the *FCGR2/3* cluster in mammals. Bioinformatic analysis of orthologous genes of human *FCGR2A, FCGR2B*, and *FCGR3A* (blue, yellow, and purple backgrounds, respectively). For gorilla and chimpanzee, the *FCGR2/3* cluster is characterized by additional human orthologous *FCGR2C* and *FCGR3B* genes. Similarly, orthologous genes of human *HSPA6* (red background) were found in all studied species whereas only gorilla and chimpanzee possess human orthologous *HSPA7*. **(B)** Non allelic homologous recombination between *FCGR2A* and *FCGR2B* leading to duplication of *FCGR4* (giving birth to *FCGR3B* and *FCGR3A*) and emergence of *FCGR2C*.

Sequences drawn from the Chimpanzee Genome Project were reassembled using the human *FCGR2/3* cluster as reference. It appears that the chimpanzee's cluster comprises orthologs of all the human *FCGR* genes as well as of the *HSPA6* and *HSPA7* genes. However, it should be noted that, through this approach, the *FCGR2C* gene could not be unambiguously assigned. Similarly, we analyzed the *FCGR2/3* cluster in macaque (*Macaca mulatta*) using sequences from Rhesus Macaque Genome Resources. We found traces of sequences that suggest that macaque cluster is composed by only three *FCGR* genes and one *HSPA6* gene (*FCGR2A*-*HSPA6*-*FCGR4*-*FCGR2B*) corresponding to orthologous genes of human *FCGR2A, HSPA6, FCGR3A*, and *FCGR2B*. However, based on the available sequences and reassembled contigs, it cannot be totally excluded that *FCGR3B* and *FCGR2C* genes do exist in this species. Sequences available for rat, cow and partial sequence from dog revealed a genomic organization close to that of mouse cluster which demonstrates that *fcgr3-hspA6fcgr4-fcgr2b* is the standard cluster for non-primate mammals (Figure 2A). Close inspection of the whole human cluster allowed us to define two duplication modules of approximately ~80 kb with the same architecture, on either side of the breaking point region already associated with *FCGR2C* genesis (9). The upstream module (b1), from *FCGR2A* to *FCGR2C*, encompasses *HSPA6* and *FCGR3A*, whereas the downstream module (b2), from *FCGR2C* to *FCGR2B*, encompasses *HSPA7*, and *FCGR3B* (Figure 2A). The b1 and b2 modules are colinear with a remarkable identity score (94%), raising the hypothesis of a tandem duplication of the b module in a former *FCGR2A*-*FCGR3*-*HSPA6*-*FCGR2B* cluster, which will be referred to as *FCGR2A*-*FCGR4*-*HSPA6*-*FCGR2B* with respect to the nomenclature already in use in other species. This event was simultaneously responsible for the duplication of *FCGR4* (which subsequently evolved in *FCGR3A* and *FCGR3B*), *HSPA6* (duplicated in *HSPA6* and *HSPA7*), and for the emergence of *FCGR2C*, made of the 5′ part of *FCGR2B* and of the 3′part of *FCGR2A* (Figure 2B).

#### PCR Screening for Orthologous Genes of Human FCGR2 and FCGR3

**Figure 3:**
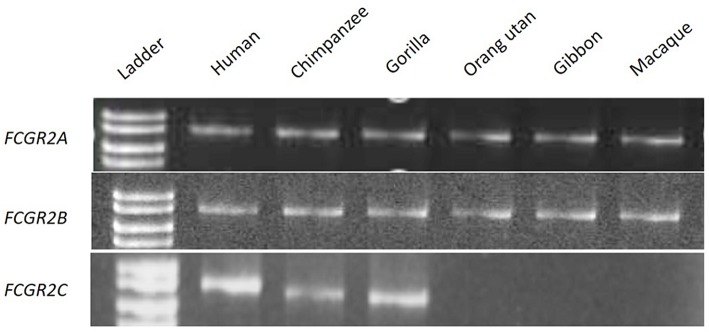
PCR screening for orthologous genes of *FCGR2A, FCGR2B*, and *FCGR2C*.

We were then interested to know whether the intermediary species between Cercopithecids (macaques and baboons) and Hominids (chimpanzees and humans), i.e., Hylobates and Pongids, possess both the *FCGR2C* and *FCGR3B* genes, or not. In this purpose, two primer pairs were first designed to specifically amplify *FCGR2A* and *FCGR2B* whatever the primate species, in a representative set of Old World primates. Using the *FCGR2B*-specific forward and the *FCGR2A*-specific reverse primers, *FCGR2C* is specifically amplified if present (Supplementary Figure 1). An amplification product was obtained with human, chimpanzee and gorilla DNA, but not with Cercopithecinae (Macaque), Hylobates (Gibbon) and Pongids (orang-utan) DNAs, suggesting an absence of *FCGR2C* (Figure 3) in these species. For these three *FCGR2* genes, and for all the tested primate species, gene specificity of the amplification products was confirmed by sequence conservation and organism specificity was controlled by sequence divergence observed at PCR product end.

Similarly, a PCR was designed to co-amplify *FCGR3B* together with *FCGR3A*. *FCGR3B* is characterized by a serine at codon position 203 (Ser^203^ generating the glycosyl-phosphatidylinositol anchor of FcγRIIIB) and a stop codon at position 216. The co-amplification product of *FCGR3A* and *FCGR3B* of human genomic DNA reveals a T (Phe) or a C (Ser) at the second position of codon 203 or a C (Arg) and T (stop) at the first position of codon 216. Both types of codons were observed at each position in gorilla and chimpanzee DNA, whereas only a Phe^203^ and an Arg^216^ or an alternative Pro^216^ codon were found in macaque, gibbon and orang-utan (Supplementary Figure 2). These results were confirmed with a different priming strategy. Two *FCGR3B* specific primers sets obtained by substitution of one of the aforementioned pair of primers by another one anchored on *FCGR3B* specific sequences failed to give amplification products in the macaque, gibbon and orang-utan DNA, but again, were effective in amplifying *FCGR3B* in humans, chimpanzees and gorillas (data not shown).

#### Comparative Sequence and Phylogenetic Analysis

To investigate the evolutionary relationships among *FCGR2* genes, we constructed neighbor-joining trees using sequences obtained for *FCGR2A, FCGR2B*, and *FCGR2C* genes at various levels of the sequence. Using exon 3, the obtained patterns show two major groups of genes where murine *Fcgr2b* and *Fcgr3* are orthologous to primate *FCGR2B* and *FCGR2A*, respectively (Supplementary Figure 3). This approach also revealed the paralogous relationship existing between *FCGR2B* and the 5′part of *FCGR2C*. Predicted amino acid sequences obtained with exon 4 (coding for EC2) shows a high degree of conservation in primates, notably for amino acids implicated in the interaction with the Fc portion of IgG (Supplementary Figure 4). Phylogenetic analysis of exons 4, 5, and 6 showed a deviation from the predicted evolutionary relationships among the *FCGR2* genes in the different species and indicates gene conversion events (data not shown). Phylogenetic tree deduced from *FCGR3A* and *FCGR3B* sequences revealed that *FCGR3A* and *FCGR3B* are paralogous genes and that *Fcgr4* is orthologous to primate *FCGR3A* (Supplementary Figure 3B). We also observed a high degree of conservation between primate FcγRIIIA for amino acids implicated in the interaction with the Fc portion of IgGs (Supplementary Figure 5).

## Discussion

This study provides insights into the genomic structure and evolution of the *FCGR* cluster in mammals. We searched the genomes of non-human primates for low/medium affinity FCGR orthologs, amplified the corresponding genes using a long-PCR approach, characterized the products and deduced the evolutionary history of this locus between mice and humans. We conclude that a NAHR mechanism gave rise to a new block encompassing FCGR2C and FCGR3B in humans, chimpanzees and gorillas. A potential limitation of this study resides in the risk of decreased sensitivity when using a single pair of primers per gene. These long primers were designed using the screened contiguous genomes described in the methods section, to be highly gene-specific. However, they were designed in highlyconserved regions, as described in polymorphism studies performed in macaques (19). Moreover, these 2 genes were either both present or absent in the amplification products, supporting the hypothesis of a NAHR. The duplication gave rise to *FCGR2C* and two *FCGR4*-type genes that diverged from each other, *FCGR3A* keeping the original *FCGR4* functionality and *FCGR3B* acquiring specific features. This duplication event took place in homininaes, and was rendered possible by the former evolution of mammal *fcgr3* into primate *FCGR2A*. This latter evolution is presently shown to be the result of serial processes leading to the genesis of *FCGR2A* coding for the new activating receptor FcγRIIA.

The evolution from *fcgr3* in non-primate mammals, to *FCGR2A* in primates appears to be the result of two genetic events comprising a NAHR between *fcgr3* and *fcgr2b*, and the integration of a retroviral element creating a new exon coding for an ITAM-like motif, which is a unique characteristics of primate *FCGR2A*. As a consequence, primates possess a new *FCGR2A* gene encoding FcγRIIA, and their genomes display a new *FCGR2A*/*FCGR4*/*FCGR2B* locus, as compared to non-primate mammals. Several hypotheses could be raised to explain the simultaneous *fcgr2b*/*fcgr3* NAHR and endogenous retrovirus integration. In mammals, and notably in mice, the homology between *fcgr3* and *fcgr2b* is restricted to exons coding for extracellular domains. A first recombination event probably occurred between *fcgr3* and *fcgr2b* genes, bringing the EC2-IC3 *fcgr2b* gene segment into *fcgr3* and probably conferring an inhibitory function to FcγRIII since the *FCGR2B* IC3 exon encodes the ITIM motif. The subsequent retrovirus integration led to the disruption of the IC2-IC3 splicing site, suppressing IC3 transcription and hence the inhibitory function of the receptor. These observations also suggest that the retrovirus that disrupted the “inhibitory *fcgr3* gene” (*fcgr2b/3*) brought the DX_2_YX_2_LX_**11**_YX_2_L ITAM motif coding sequence.

Therefore, the loss of ITIM and the acquisition of ITAM were probably contemporaneous. Such an *fcgr2b/3* gene has never been found in living species but too few of them have been studied so far to be conclusive. We could however raise the hypothesis that the presence of two genes (*fcgr2b* and *fcgr2b/3*) encoding inhibitory FcγRs has rapidly been counter-selected unless one of them was rescued, which is now the case for *fcgr2b/3*, in the form of FCGR2A, in macaques and apes. Indeed, like a knock-in process, an endogenous retrovirus disrupted the ITIM encoding exon and brought an ITAM-like motif allowing the final transformation of *fcgr2b/3* into *FCGR2A*, the first activating FcγR not depending on the accessory FcR-γ chain. Hundreds examples of molecular domestication have been described where coding sequences of functional genes derive entirely or partially from mobile element sequences but, to our knowledge, this is the first case where a new transduction motif originates from a transposon (20). This motif has been classified among the ITAMs despite the fact that it contains 11 (rhesus macaque) or 12 (pigtailed macaque, cynomolgus macaque apes and humans) amino acids instead of 7 between the two YXXL boxes. This atypical structure is responsible for the presence of three phosphorylation sites and is associated with specific functions (21–23). This once again illustrates the power of mobile elements in driving the evolution and diversification of immunity-related genes (24).

It has long been known that the cluster that encodes low-affinity FcγRs displays different genomic structures depending on the species (25). However, the initial scenario proposed to explain its evolution amongst mammals did not integrate subsequent findings such as the mechanism of *FCGR2C* genesis, the global structure of the human cluster and the structure of the murine cluster revealing the presence of *Fcgr4* (2, 9, 25, 26). It is now clear that most mammals possess two activating and one inhibiting *fcgr* genes tighly associated with *hspa6* in an ancestral cluster (*fcgr3*-*hspa6*-*fcgr4*-*fcgr2b*) (Figure 1). The fact that the murine cluster consists of only 3 *fcgr* genes appears as an exception rather than a rule, suggesting that *hspa6* has been lost in the laboratory strains of mice. Monkeys also possess a four-gene cluster, differing from the ancestral cluster by the presence of *FCGR2A* rather than *fcgr3*. It has been shown that the human *FCGR2/3* cluster is composed of two repetition blocks (∽80 kb each with > 95 % homology) which results from a segmental duplication, according with its current definition (27). *FCGR2C*, a well-known chimeric gene, links the two modules and appears as a signature of the segmental duplication event (9). This recombination event is the result of an unequal cross-over favored by the high degree of homology between *FCGR2A* and *FCGR2B*. Since *FCGR2C* can be found in gorillas and chimpanzees, contrarily to what had been previously published by Nimmerjahn et al. but not in orangutans, gibbons and macaques, the duplication event probably occurred in a homininae ancestor <9.2 million years ago (7, 28). Although the duplication gave rise to two *FCGR4* copies, *FCGR3B* was identified in each species where *FCGR2C* was found. This suggests that the second copy of *FCGR4* rapidly evolved into *FCGR3B*, i.e., into a gene coding for the only known GPIanchored FcγR.

It is known that the presence of large duplicated segments subsequently favor additional NAHR events (29–31) resulting in gene number variations. A variable number of *FCGR2C* and *FCGR3B* was described and it seems that a co-variation number of these genes occurred in most cases (32–34). This co-variation of *FCGR2C*/*FCGR3B* copy number described in humans could be explained by the modular structure of the cluster which favors further recombination events through unequal crossing over between clusters, involving *FCGR2C* and upstream sequence on one chromatide, and *FCGR2B-*related sequences on the other (Supplementary Figure 6). Following this recombination model, human haplotypes characterized by *FCGR3B* duplication would also contain two *FCGR2C* and two *HSPA7*. By contrast, haplotypes characterized by an absence of *FCGR3B* would also be deficient in *FCGR2C* and *HSPA7*. As a matter of fact, a 10-fold higher identity region can be observed between modules b1 and b2 in the region expected to be place of secondary segmental duplication.

Altogether, it is clear that the low-affinity Fc receptor gene cluster has been submitted to serial genetic events which have been positively selected during primate evolution. In comparison with other mammals, homininaes -comprising humans- have acquired FcγRIIA, FcγRIIC, and FcγRIIIB. At least two of these receptors are fully functional (FcγRIIA and FcγRIIIB) and are involved in the mechanism of action of therapeutic antibodies. Finally, our results highlight genomic differences between humans and other mammals in terms of FcγR coding genes. Additional differences in terms of FcγR expression and function raise the issue of the relevance of animal models for pre-clinical studies of monoclonal antibodies.

## Author Contributions

GB reanalyzed the data and provided critical revision and updating of the manuscript. JL performed the experiments, analyzed the results, and wrote the first draft of the manuscript. HW supervised the experiments and provided expert corrections to the manuscript.

### Conflict of Interest Statement

The authors declare that the research was conducted in the absence of any commercial or financial relationships that could be construed as a potential conflict of interest.

## Abbreviations

ADCC: antibody-dependent cell mediated cytotoxicity
EC: extracellular domain
ECACC: European collection of authenticated cell cultures
FcγR: Fc-gamma receptor
IC: intracellular domain
ITAM: immunoreceptor tyrosine-based activation motif
ITIM: immunoreceptor tyrosine-based inhibition motif
LTR: Long terminal repeat sequence
NAHR: non-allelic homologous recombination
NHP: non-human primate
PCR: polymerase chain reaction.
