# Supplementary material for: Evolutionary Story of the Low/Medium-Affinity IgG Fc Receptor Gene Cluster
Source: Front Immunol. 2019 Jun 6;10:1297. doi: 10.3389/fimmu.2019.01297 (PMC6563257; doi:10.3389/fimmu.2019.01297)
Supplement: Supplementary file 1 [file Data_Sheet_1.docx]

Evolutionary story of the low/medium-affinity IgG Fc receptor gene cluster: supplementary information

**
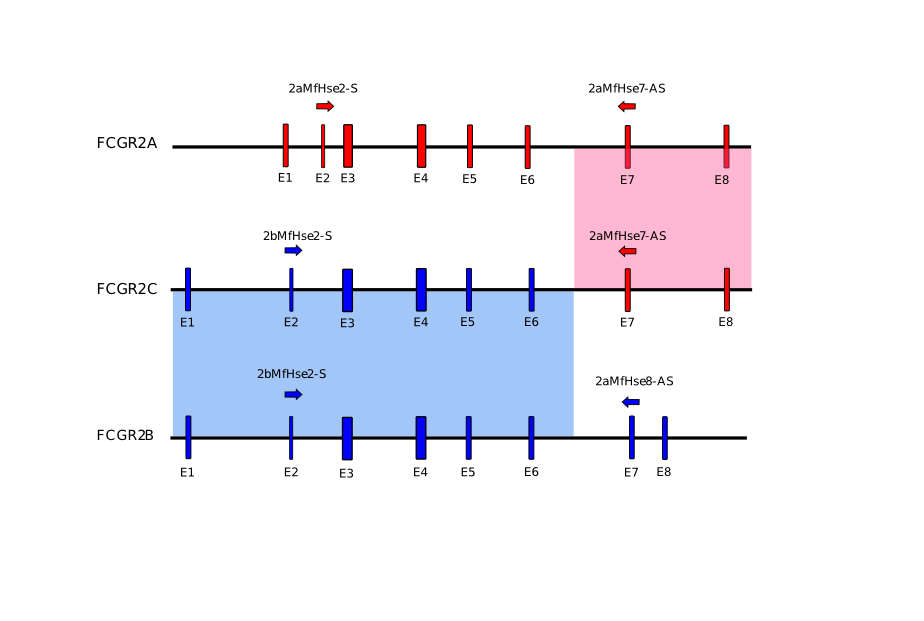
Supplementary Figure 1. Long PCR approach and primers used for *FCGR2A*, *FCGR2B* and *FCGR2C* screening.**

**Supplementary Figure 2: PCR screening for orthologous genes of FCGR2A, FCGR2B and FCGR2C.**

**A.** Co-amplification of FCGR3A and FCGR3B **B.** Specific PCR for FCGR3B. **D.** Sequencing of co amplification product revealed presence of FCGR3A and FCGR3B in gorilla whereas only codon specific for FCGR3A is observed for Oran-utan.


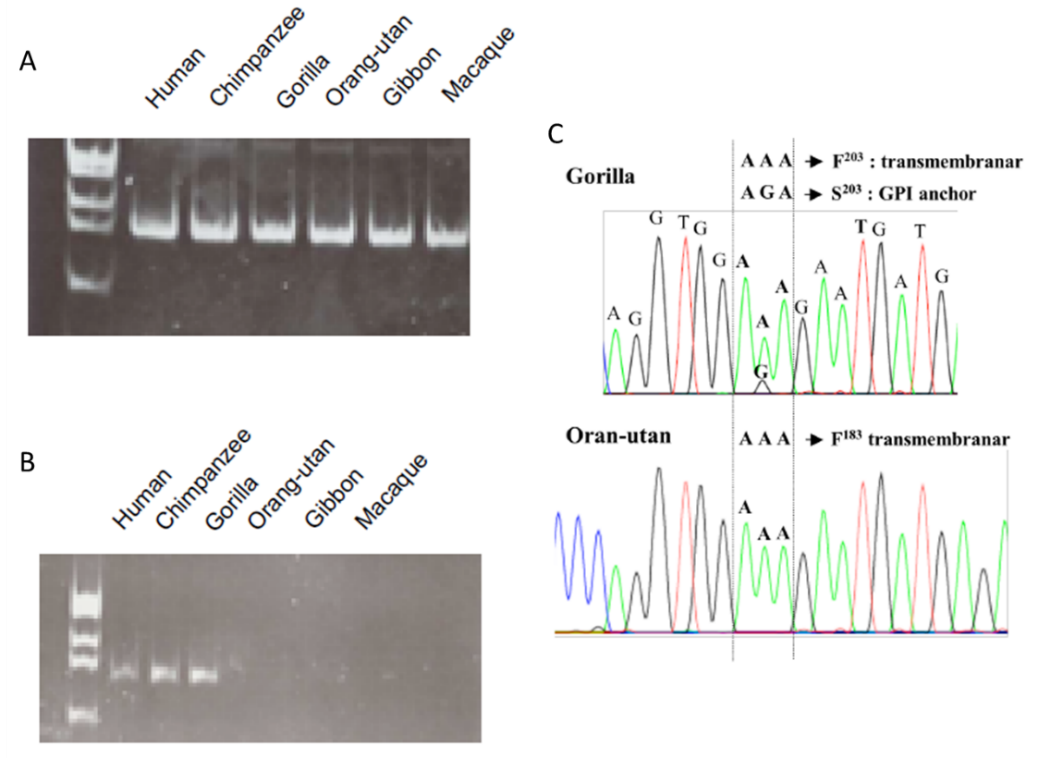


**Supplementary Figure 3**

Neighbor-joining trees of FCGR2/3 genes (A and B respectively). Phylogenetic analysis was carried out in human (Homo sapiens; Hs), chimpanzee (Pan troglodytes; Pt), gorilla (Gorilla gorilla, Gg), orangutan (Pongo pygmaeus; Pp), Gibbon (Hylobate iar, Hi), macaque (Macaca fascicularis, Mf) and in mouse (Mus musculus, Mm).

**
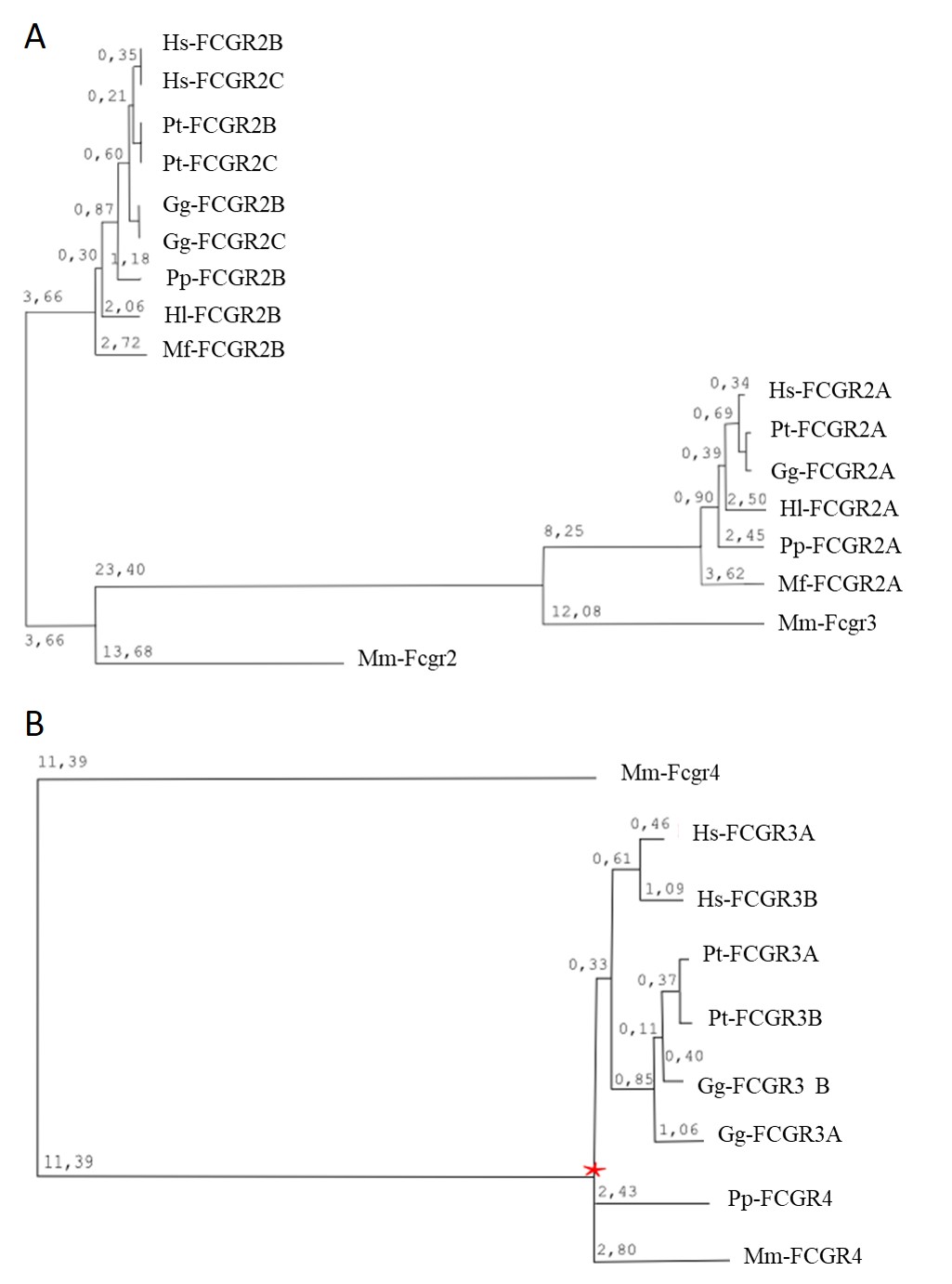
**


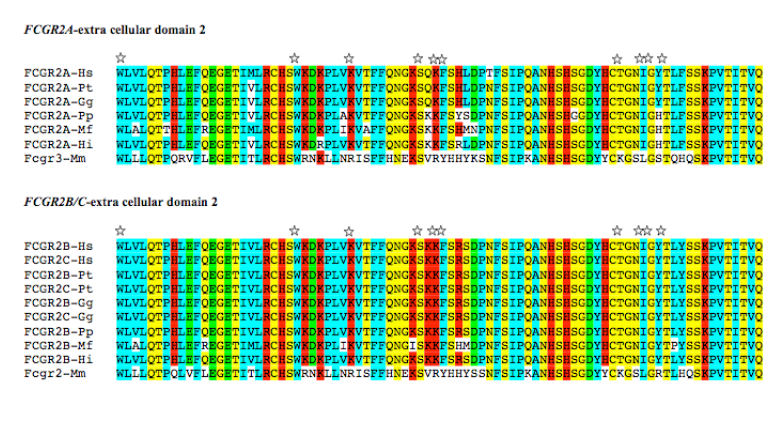
**Supplementary Figure 4: Predicted amino acid sequences of extra-cellular domain 2 of FCGR2A/2B.** human (Homo sapiens ; Hs), chimpanzee (Pan troglodyte ; Pt), gorilla (Gorilla gorilla , Gg), orangutan (Pongo pygmaeus ; Pp), Gibbon (Hylobate iar, Hi), macaque (Macaca fascicularis, Mf) and in mouse (Mus musculus, Mm).


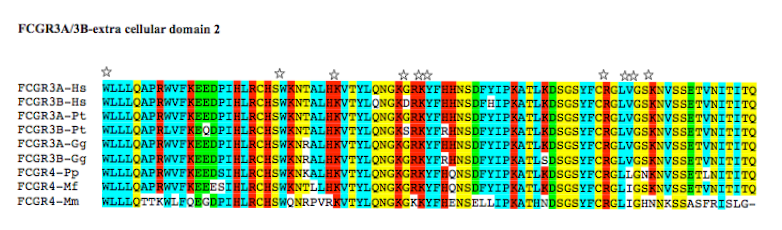
**Supplementary Figure 5:** **Predicted amino acid sequences of extra-cellular domain 2 of FCGR3A/3B.** human (Homo sapiens; Hs), chimpanzee (Pan troglodyte; Pt), gorilla (Gorilla gorilla, Gg), orangutan (Pongo pygmaeus; Pp), macaque (Macaca fascicularis, Mf) and in mouse (Mus musculus, Mm).

**Supplementary Figure 6**

Recombination model inside the FCGR locus explaining the covariation of FCGR2C/FCGR3B copy number.

**
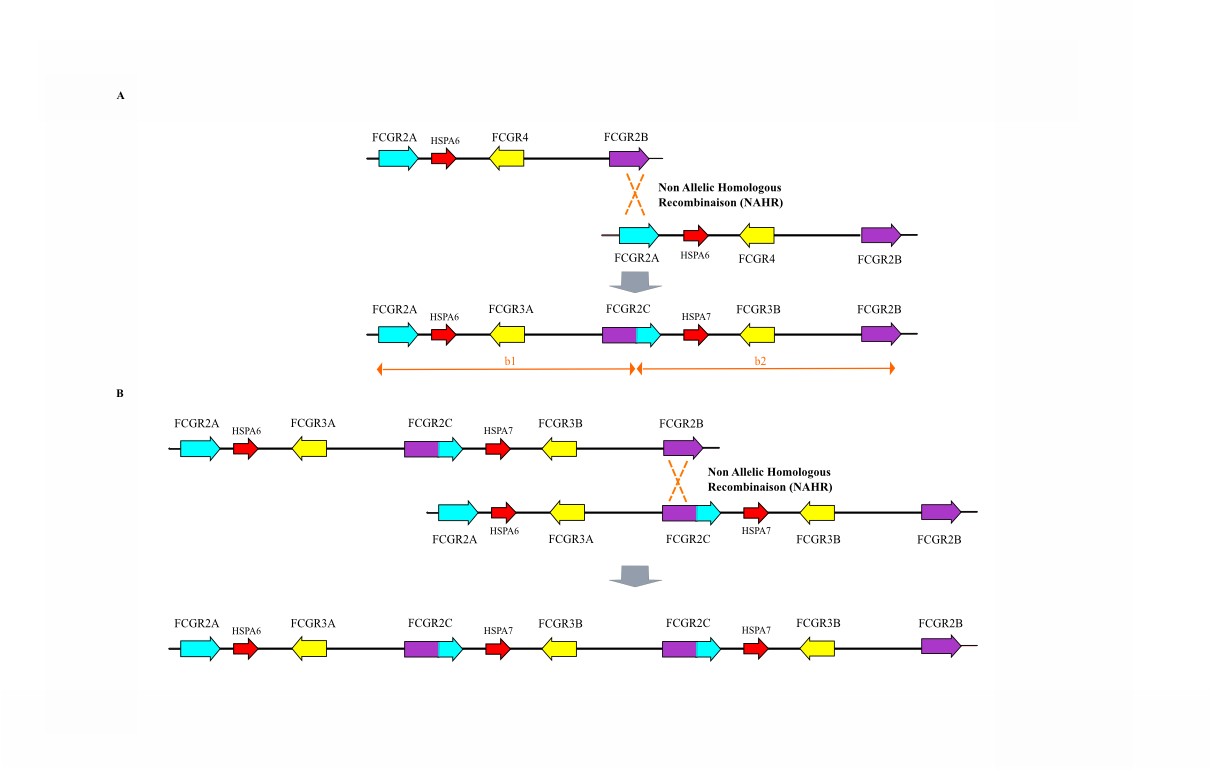
**

**Supplementary table 1: genome versions used and comparison with actual versions**

| **Species** | **Sequence name** | **Used version** | **Actual version** |
| --- | --- | --- | --- |
| **Human** | **RP11-5K23** | **AL590385.23** | **AL590385.23** |
| **Human** | **RP11-25K21 contig a** | **AL451067.10** | **AL451067.12** |
| **Human** | **RP11-25K21** | **AL451067** | **AL451067.12** |
| **Human** | **RP11-25I17** | **AC021370.4** | **AC021370.4** |
| **Human** | **RP11-25K21 contig b** | **AL451067.1** | **AL451067.12** |
| **Human** | **RP11-247I16** | **AL359541** | **AL359541.11** |
| **Pan troglodytes** | **AACZ01374181 to -8** | **Cont2734** | **AACZ00000000.4** |
| **Pan troglodytes** | **AADA01327254** | **ctg_327253** | **AADA01327254.1** |
| **Pan troglodytes** | **AACZ01050582 to -6** | **Cont3131** | **AACZ00000000.4** |
| **Pan troglodytes** | **AADA01001879** | **ctg_1878** | **AADA01001879.1** |
| **Pan troglodytes** | **AADA01295462** | **ctg_295461** | **AADA01295462.1** |
| **Pan troglodytes** | **AACZ01194036 to -40** | **Cont7248** | **AACZ00000000.4** |
| **Macacca mulatta** | **NW_001108971** |  | **NW_001108971.1** |
| **Mus musculus** | **NC_000067** |  | **NC_000067.6** |
| **Canis lupus** | **NW_876305** |  | **NW_876305.1** |
| **Bos taurus** | **NW_931054** |  | **NW_931054.1** |
| **Rattus norvegicus** | **NC_005112** |  | **NC_005112.4** |

**Supplementary Table 2**

**Primers used for sequencing of *FCGR2A*, *FCGR2B* and *FCGR2C*.**

**Supplementary table 3**

**Annotation of the retroelement contained in the human fcgr locus using ReapeatMasker**
